# Sorafenib increases cytochrome P450 lipid metabolites in patient with hepatocellular carcinoma

**DOI:** 10.3389/fphar.2023.1124214

**Published:** 2023-03-03

**Authors:** Can G. Leineweber, Miriam Rabehl, Anne Pietzner, Nadine Rohwer, Michael Rothe, Maciej Pech, Bruno Sangro, Rohini Sharma, Chris Verslype, Bristi Basu, Christian Sengel, Jens Ricke, Nils Helge Schebb, Karsten-H. Weylandt, Julia Benckert

**Affiliations:** ^1^ Medical Department B, Division of Hepatology, Gastroenterology, Oncology, Hematology, Palliative Care, Endocrinology, and Diabetes, Brandenburg Medical School, University Hospital Ruppin-Brandenburg, Neuruppin, Germany; ^2^ Faculty of Health Sciences, Joint Faculty of the Brandenburg University of Technology, Brandenburg Medical School and University of Potsdam, Potsdam, Germany; ^3^ Institut d’Investigacions Biomèdiques August Pi i Sunyer (IDIBAPS), Barcelona, Spain; ^4^ Department of Molecular Toxicology, German Institute of Human Nutrition Potsdam-Rehbruecke, Nuthetal, Germany; ^5^ Lipidomix, Berlin, Germany; ^6^ Department of Radiology and Nuclear Medicine, Otto-von-Guericke University, Magdeburg, Germany; ^7^ Liver Unit and HPB Oncology Area, Clinica Universidad de Navarra and CIBEREHD, Pamplona, Spain; ^8^ Department of Surgery and Cancer, Imperial College London, London, United Kingdom; ^9^ Department of Digestive Oncology, University Hospitals Leuven, Leuven, Belgium; ^10^ Department of Oncology, University of Cambridge, Cambridge, United Kingdom; ^11^ Radiology Department, Grenoble University Hospital, La Tronche, France; ^12^ Department of Radiology, University Hospital, Ludwig-Maximilians-University (LMU) Munich, Munich, Germany; ^13^ Chair of Food Chemistry, Faculty of Mathematics and Natural Science, University of Wuppertal, Wuppertal, Germany; ^14^ Department of Hepatology and Gastroenterology, Charité—Universitätsmedizin Berlin, Corporate Member of Freie Universität Berlin and Humboldt—Universität zu Berlin, Berlin, Germany

**Keywords:** hepatocellular carcinoma, cytochrome P450, sorafenib, EET, EDP, omega-3 fatty acids, oxylipins, lipidomics

## Abstract

Hepatocellular carcinoma (HCC) is a leading cause of cancer death, and medical treatment options are limited. The multikinase inhibitor sorafenib was the first approved drug widely used for systemic therapy in advanced HCC. Sorafenib might affect polyunsaturated fatty acids (PUFA)-derived epoxygenated metabolite levels, as it is also a potent inhibitor of the soluble epoxide hydrolase (sEH), which catalyzes the conversion of cytochrome-P450 (CYP)-derived epoxide metabolites derived from PUFA, such as omega-6 arachidonic acid (AA) and omega-3 docosahexaenoic acid (DHA), into their corresponding dihydroxy metabolites. Experimental studies with AA-derived epoxyeicosatrienoic acids (EETs) have shown that they can promote tumor growth and metastasis, while DHA-derived 19,20-epoxydocosapentaenoic acid (19,20-EDP) was shown to have anti-tumor activity in mice. In this study, we found a significant increase in EET levels in 43 HCC patients treated with sorafenib and a trend towards increased levels of DHA-derived 19,20-EDP. We demonstrate that the effect of sorafenib on CYP- metabolites led to an increase of 19,20-EDP and its dihydroxy metabolite, whereas DHA plasma levels decreased under sorafenib treatment. These data indicate that specific supplementation with DHA could be used to increase levels of the epoxy compound 19,20-EDP with potential anti-tumor activity in HCC patients receiving sorafenib therapy.

## 1 Introduction

Liver cancer is a global issue, being the most common cancer and the leading cause of cancer death in transition countries. In 2020, almost 9,06,000 patients were diagnosed with liver cancer and over 8,30,000 deaths were documented worldwide. Hepatocellular carcinoma (HCC) has the highest prevalence among the different subtypes of liver cancer (Sung et al., 2021). Viral infections, more specifically hepatitis B and C virus (Datfar et al., 2021), lifestyle factors such as alcohol intake (Matsushita and Takaki, 2019), as well as type 2 diabetes and non-alcoholic fatty liver disease (NAFLD) (Estes et al., 2018) remain the leading risk factors, depending on the region considered. Hepatocellular carcinoma remains one of the most common causes of cancer death, especially in men, and has one of the lowest 5-year survival rates of all different cancer types (Siegel et al., 2022).

In addition to chronic inflammation, tissue remodeling and changes in cellular signaling are pathogenetic factors of carcinogenesis (Refolo et al., 2020). Interestingly, patients with a non-cirrhotic HCC, mostly caused by NAFLD, seem to show a more severe HCC histopathology on one hand but better overall survival on the other hand (Gawrieh et al., 2019).

Limited stages of HCC can be treated with locoregional procedures such as surgical (e.g., resection, transplantation) or radiological (e.g., transarterial chemoembolization, selective internal radiotherapy) intervention, prolonging survival for more than 5 years, depending on the underlying liver disease. Once the disease progresses, survival is compromised to approximately 1 year even with systemic therapy (Forner et al., 2018; Vogel et al., 2022). Systemic therapy options were shown to increase overall survival (OS) and progression-free survival (PFS). However, either applying combination therapies including immune checkpoint inhibitors such as atezolizumab plus bevacizumab (OS 13,4-19,2 months; PFS 6,9 months) (Finn et al., 2020) or tyrosine kinase inhibitors lenvatinib (OS 13,6 months; PFS 7,3 months) (Kudo et al., 2018) or sorafenib (OS 10,7-15,5 months; PFS 3,6-5,5) (Kelley et al., 2022) has not altered the severity or mortality of the disease so far (Vogel et al., 2022).

The first targeted and currently a widely used systemic therapy for HCC is the oral multikinase inhibitor sorafenib binding in an ATP-binding pocket to inhibit kinase function (Hwang et al., 2013), which predominantly inhibits angiogenesis *via* binding the vascular endothelial growth receptor (VEGFR). Furthermore, it targets the cell proliferation and differentiation *via* the rapidly accelerated fibrosarcoma (RAF) signaling pathway (Wilhelm et al., 2004) and the platelet-derived growth factor receptor-β (PDGFR-β) (Mody and Abou-Alfa, 2019) as well as the beneficial effects, particularly in cancer and its complications, which is likely due to the inhibition of nuclear factor kappa B (NF-κB) and production of the pro-resolution mediators at the molecular level (Freitas and Campos, 2019).

In addition to the known anti-angiogenic and anti-proliferative effects of sorafenib it has also been described to have effects on the soluble epoxide hydrolase (sEH) which showed similar anti-inflammatory effects as conventional sEH inhibitors in lipopolysaccharide-induced inflammation models in mice (Liu et al., 2009). Sorafenib is a potent inhibitor of sEH compared with conventional urea-based sEH inhibitors (Hwang et al., 2013). The sEH is expressed in numerous human tissues with the main distinction between microsomal epoxide hydrolase (mEH) and sEH (Morisseau and Hammock, 2013). The sEH metabolization is the dominant pathway in humans, so that it can be assumed that sEH inhibition has a stabilizing effect on endogenous epoxy metabolites in tissues (Spector and Kim, 2015).

**FIGURE 1:**
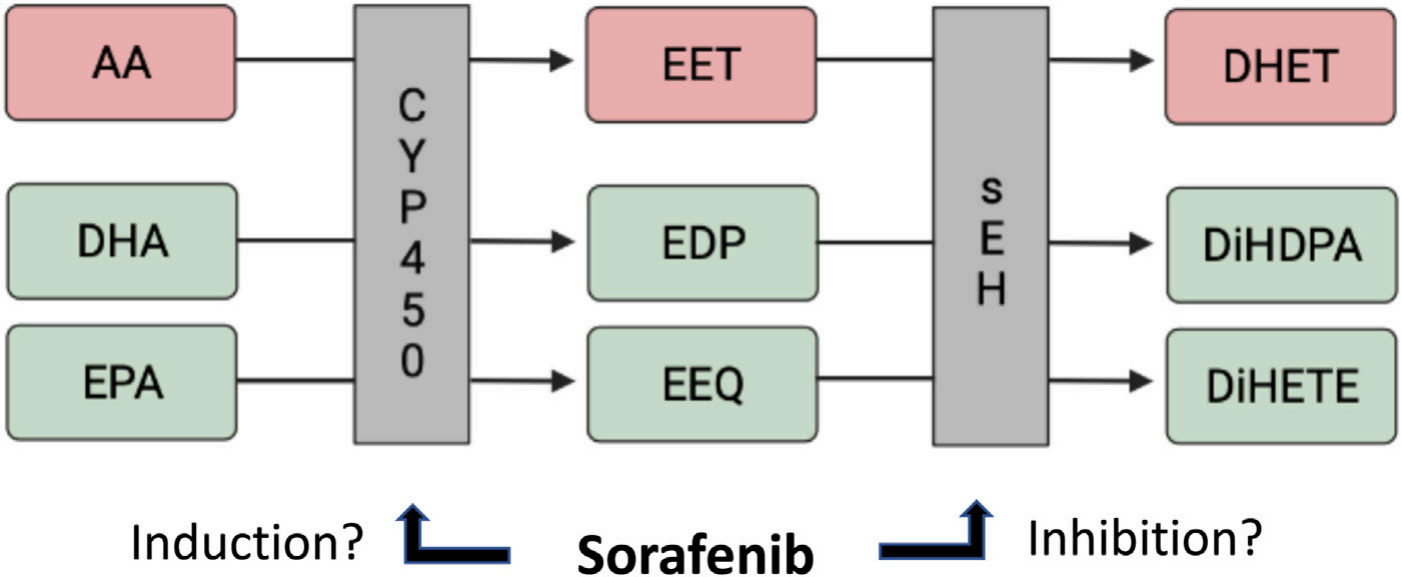
CYP-dependent lipid metabolite formation from AA/DHA/EPA, and the potential effects of sorafenib.

The epoxidation of long-chain polyunsaturated fatty acids (LC-PUFAs), such as the omega-6 (n-6) PUFA arachidonic acid (C20:4n6, AA), as well as the omega-3 (n-3) PUFAs eicosapentaenoic acid (C20:5n3, EPA) and docosahexaenoic acid (C22:6n3, DHA) to epoxyeicosatrienoic acids (EETs), epoxyeicosatetraenoic acids (EEQs) and epoxydocosapentaenoic acids (EDPs), respectively, are catalyzed by the cytochrome P450 (CYP) epoxygenases (Figure 1). These epoxymetabolites are then further metabolized *via* sEH into their biologically less active corresponding dihydroxy metabolites (Zhang et al., 2014; Wang et al., 2018).

The role of epoxidized LC-PUFAs is well established in several biological processes, such as angiogenesis, inflammation, and tumor growth: In different animal models, it has been shown that sEH-inhibition has a positive influence on cardiovascular and liver abnormalities (Iyer et al., 2012), liver fibrosis and portal hypertension (Zhang et al., 2018), fatty liver (Yao et al., 2019) and non-alcoholic steatohepatitis (Wang et al., 2019). The EETs are known to affect blood pressure, inflammation, pain sensation, and regeneration (Arnold et al., 2010; Morisseau et al., 2014). However, a proangiogenic effect of 11,12-EET and 14,15-EET, the main EET regioisomers in mammals (Spector and Kim, 2015), has been described *via* the epidermal growth factor (EGF) and VEGF pathways (Zhang et al., 2014), which may explain the finding that EETs promote tumor growth. The n-6 AA-derived regioisomers 5,6-EET and 8,9-EET were found to increase cell proliferation and *de novo* vascularization (Yan et al., 2008), whereas 11,12-EET and 14,15-EET promote tumor angiogenesis through endothelial cell proliferation (Panigrahy et al., 2012; Zhang et al., 2013). An increase in 14,15-EET through sEH-inhibition led to increased tumor growth and metastasis through cell invasion in experimental studies (Panigrahy et al., 2012). In summary, through mechanisms of cell proliferation, *de novo* vascularization and endothelial proliferation several EET-regioisomers promote tumor growth and metastasis (Yan et al., 2008; Panigrahy et al., 2012; Zhang et al., 2013; Zhang et al., 2014). The sEH-inhibitory effect of sorafenib might thus carry clinically relevant consequences by increasing these pro-tumorigenic EET mediators.

In contrast, n-3 PUFA-derived regioisomers show anti-angiogenic effects both *via* the VEGF and FGF-2 pathway (Zhang et al., 2014). In a tumor mouse model, it was shown, that low dose sEH-inhibition led to an increase in n-3 DHA-derived 19,20-EDP and thereby reduced tumor angiogenesis and cell invasion and thus inhibition of tumor growth (Zhang et al., 2013). Furthermore, a protective effect in obesity and obesity-related comorbidities, such as fatty liver disease, of n-3 epoxy PUFA was found in animal models (López-Vicario et al., 2015). The beneficial effects of n-3 PUFA regarding to cancer and its complications are probably due to their anti-inflammatory and pro-resolution mediators (Freitas and Campos, 2019). In the context of abundant DHA, the sEH-inhibitory effect of sorafenib might thus lead to higher levels of 19,20-EDP, mediating anti-tumor effects.

Currently, n-6 PUFA are found in a ratio of approximately 20 times more than n-3 PUFA in the human diet (Harris, 2006); therefore humans have low n-3 PUFA tissue levels, and a shift of the competitive n-6 and n-3 PUFA metabolism towards n-6 PUFA derived lipid metabolites (Spector and Kim, 2015).

We therefore aimed to investigate levels of n-6 and n-3 PUFA in HCC patients, as well as n-6 PUFA- and n-3 PUFA-derived epoxide and corresponding dihydroxy compounds in HCC patients without and during sorafenib therapy. Based on the data of our pilot study (Leineweber et al., 2020), we hypothesized that sorafenib treatment, due to sEH-inhibitory and possibly CYP-modulating effects, might increase the presence of potentially tumor growth-suppressing DHA-derived EDPs, as well as of potentially tumor growth-promoting EETs.

## 2 Materials and methods

### 2.1 Patients and blood sampling

The study population evaluated in this sub-analysis comprised patients within the palliative treatment arm of the randomized, controlled, multicenter phase II SORAMIC study, which evaluated sorafenib alone compared to selective internal radiation therapy (SIRT) combined with sorafenib on overall and progression-free survival in patients with advanced HCC (Ricke et al., 2019).

Patients were included in this analysis if they received study treatment in the palliative arm of SORAMIC and signed an informed consent form, so blood samples were collected and analyzed at baseline (BL) and at the first follow-up visit (FU) after approximately 7–9 weeks, and samples were stored for subsequent analyses at −80°C. Of the 424 randomized patients assigned to the palliative arm, we performed lipidomic analysis of 43 patients from the intention to treat (ITT) population, all of whom received sorafenib, characterized in terms of gender distribution, age, body mass index (BMI), presence of cirrhosis, liver function, biomarker, or tumor stage according to Barcelona Clinic Liver Cancer (BCLC) stage, except in the expression of the Child-Pugh points between 5 and 6/7 (*p* < 0.0453) as shown in Table 1.

**TABLE 1:** Patient characteristics of the *n* = 43 HCC patients receiving sorafenib treatment. Data are presented as mean ± standard error of the mean.

Characteristic	Total (*n* = 43)
Gender	
Female	5 (12%)
Male	38 (88%)
Age (years)	66.97 ± 2.19
BMI	26.98 ± 0.99
Liver disease	
Alcohol	17 (40%)
Hepatitis B/C	15 (35%)
Liver cirrhosis	36 (84%)
Child Pugh	
5	28 (65%)
6/7	15 (35%)
BCLC	
B	16 (37%)
C	27 (63%)
Further disease classification	
Liver dominant disease	41 (95%)
Portal vein invasion	16 (37%)
Extrahepatic metastases	8 (19%)
Bilirubin (µmol/L) at baseline	15.91 ± 1.66
Albumin (g/L) at baseline	37.68 ± 1.42
AlBi Score at baseline	−2.44 ± 0.13
DeRitis quotient	1.65 ± 0.26
AFP	
<400 ng/mL	25 (58%)
>400 ng/mL	16 (37%)
Sorafenib: Daily dose (mg)	410.82 ± 23.40
Overall survival (months)	12.04 ± 1.46

### 2.2 Sample preparation and GC

Plasma samples were analyzed for determination of fatty acids using the gas chromatography (GC) technology as described previously (Wang et al., 2022). 75 μL of EDTA plasma per sample was used for the GC preparation. Methylation and extraction of FAs were carried out on the basis of an established protocol (Kang and Wang, 2005). Briefly, frozen samples were thawed at room temperature. All samples were then mixed with 50 μL pentadecanoic acid (PDA, 1 mg/mL in ethanol, Merck Schuchardt OHG, Hohenbrunn, Germany) as internal standard, 500 μL borontrifluoride (BF_3_, Sigma-Aldrich Chemie GmbH, Taufkirchen, Germany) in 14% methanol (Merck KGaA, Darmstadt, Germany), and 500 μL *n*-hexane (Merck KGaA, Darmstadt, Germany) in glass vials and were tightly closed. After vortexing, samples were incubated for 60 min in a preheated block at 100°C. After cooling down to room temperature, the mixture was added to 750 μL water, vortexed, and extracted for 4 min. Then all samples were centrifuged for 5 min (RT, 3,500 rpm). From each sample, 100 μL of the upper n-hexane layer was transferred into a micro-insert (placed in a GC glass vial), tightly closed, and analyzed by GC.

GC was performed on a 7890B GC System (Agilent Technologies, Santa Clara, CA, United States) with an HP88 Column (112/8867, 60 m × 0.25 mm × 0.2 μm, Agilent Technologies, Santa Clara, CA, United States), with the following temperature gradient: 50°C–150°C with 20°C/min, 150°C–240°C with 6°C/min, and 240°C for 10 min (total run time 30 min). Nitrogen was used as carrier gas (constant flow 1 mL/min). 1 μL of each sample was injected into the injector (splitless injection, 280°C). The flame ionization detector (FID) analysis was performed at 250°C with the following gas flows: hydrogen 20 mL/min, air 400 mL/min, and make up (nitrogen) 25 mL/min. Methylated FAs in the samples were identified by comparing the retention times with those of known methylated FAs of the Supelco^®^ 37 FAME MIX standard (CRM47885, Sigma Aldrich, Laramie, WY, United States) and a mix of single FAME standards [DPA, C22:5 n-3, AdA, C22:4 n-6 (Cayman Chemicals, Ann Arbor, MI, United States)]. Analysis and integration of the peaks were carried out with OpenLAB CDS ChemStation Edition (Agilent Technologies, Santa Clara, CA, United States). FA values are presented as percentage (Ramsay et al., 2018) of total FA content and absolute concentrations (µg/mL). For the study, 16 FAs were included as follows: myristic acid (C14:0), palmitic acid (C16:0), stearic acid (C18:0), arachidic acid (C20:0), behenic acid (C22:0), lignoceric acid (C24:0), palmitoleic acid (C16:1 n-7c), oleic acid (C18:1 n-9c), nervonic acid (C24:1 n-9), eicosapentaenoic acid (EPA, C20:5 n-3), docosapentaenoic acid (DPA, C22:5 n-3), docosahexaenoic acid (DHA, C22:6 n-3), linoleic acid (LA, C18: 2 n-6), dihomo-gamma- linolenic acid (DGLA, C20:3 n-6), arachidonic acid (AA, 20:4 n-6), and adrenic acid (AdA, C22:4 n-6).

### 2.3 Sample preparation and LC/ESI-MS/MS

Plasma samples were analyzed for epoxymetabolites using the LC/ESI-MS/MS lipidomics technology as described previously (Fischer et al., 2014). Lipid mediators and deuterated standards used in this study were purchased from Cayman Chemical (Ann Arbor, MI, United States). Materials used for solid phase extraction (SPE), such as sodium acetate, ethyl acetate, acetic acid, and n-hexane were obtained from Fisher Scientific (Loughborough, UK). Additionally, 99% butylated hydroxytoluene (BHT, 2,6-di-tert-butyl-4-methylphenol) was obtained from Acros Organics (Geel, Belgium), and Bond Elute Certify II columns from Agilent Technologies (Santa Clara, CA, United States) were used. LC-MS solvents, such as methanol (Lichrosoly hypergrade) and acetonitrile (Lichrosoly hypergrade), were obtained from Merck (Darmstadt, Germany).

For sample preparation, an internal standard consisting of 14,15-DHET-d11, 15-HETE-d8, 20-HETE-d6, 8,9-EET-d11, 9,10-DiHOME-d4, 12,13-EpOME-d4, 13-HODE-d4 and LTB4-d4 (500 pg each) and ice-cold methanol containing BHT (0.1%) was added to 200 mL plasma. After alkaline hydrolysis using 1 mmol sodium hydroxide the pH was adjusted with acetic acid and sodium acetate buffer containing 5% v/v methanol at pH 6. After centrifugation, the obtained supernatant was added to SPE columns, which were preconditioned with 3 mL methanol, followed by 3 mL of 0.1 mol/L sodium acetate buffer containing 5% methanol (pH 6). The SPE columns were then washed with 3 mL methanol/H2O (50/50, v/v). For elution, 2.0 mL of n-hexane:ethyl acetate (25:75) with 1% acetic acid were used. The extraction was performed with a SUPELCO Visiprep manifold. The eluate was evaporated on a heating block at 40°C under a stream of nitrogen. The solid residue was resolved in 100 µL 60% methanol in water.

The prepared samples were analyzed using an Agilent 1290 HPLC system with a binary pump, an autosampler, and a column thermostat with a Agilent Zorbax Eclipse plus C18 column 150 mm × 2.1 mm, 1.8 µm using a solvent system of aqueous acetic acid (0.05%) and acetonitrile:methanol (50:50). The multiple step elution gradient started at 95% aqueous phase, which was increased within 18 min–98% organic phase and held there for 10 min. The flow rate was set at 0.3 mL/min, and injection volume was 20 μL. The HPLC was coupled with an Agilent 6495 Triple Quad mass spectrometer with an electrospray ionization source. Analysis of lipid mediators was performed with the Multiple Reaction Monitoring in the negative mode, limit of quantitation (LOQ) was 0.01 ng/mL.

### 2.4 Statistical analysis

Statistical analysis was performed using GraphPad Prism 9 (GraphPad Software, La Jolla, CA, United States). The comparison was made using the Wilcoxon matched-pairs signed-rank test. The correlation was made using linear regression. All values are presented as the mean ± standard error of the mean. Statistical significance was assumed when *p* < 0.05. (*0.01 ≤ *p* < 0.05; **0.001 ≤ *p* < 0.01; ***p < 0.001).

## 3 Results

Blood samples from a total of *n* = 43 HCC patients were analyzed in a paired fashion with blood taken without and undergoing 7–9 weeks sorafenib treatment. This is a sub-analysis of the well-characterized study population of the randomized controlled, multicenter phase II SORAMIC trial (Ricke et al., 2019). Patients from this study population, from which suitable amounts of blood samples were available for the analysis of fatty acids and their lipid metabolites before and during sorafenib treatment, were chosen for the analysis performed and presented here. The patients received sorafenib 200 mg twice a day for 1 week before increasing the dose to 400 mg twice a day. Based on disease progression and clinical condition, the sorafenib dose was escalated to 600–800 mg or reduced to 0–200 mg. The patients’ general characteristics are shown in Table 1.

### 3.1 N-6 and n-3 epoxides and dihydroxy metabolites are higher in patients undergoing sorafenib treatment

**FIGURE 2:**
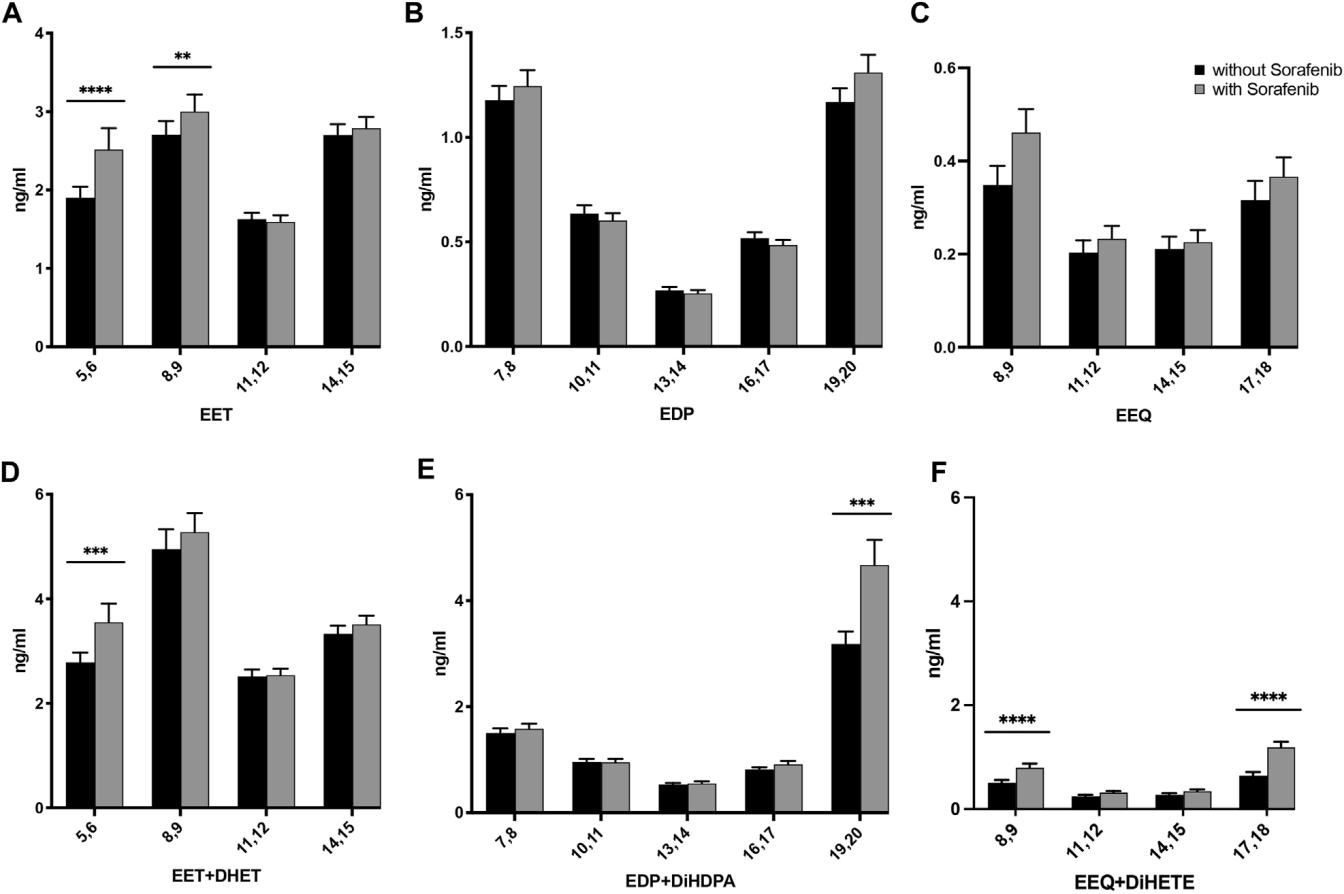
Effects on the concentrations of **(A)** AA-, **(B)** DHA-, and **(C)** EPA-derived epoxy-PUFA EETs, EDPs, and EEQs; and **(D)** AA-derived epoxy-PUFA plus dihydroxy-PUFA, **(E)** DHA-derived epoxy-PUFA plus dihydroxy-PUFA, and **(F)** EPA-derived epoxy-PUFA plus dihydroxy-PUFA in the plasma of *n* = 43 patients with hepatocellular carcinoma (HCC) without and undergoing sorafenib treatment (ng/mL ± standard error of the mean). Statistical differences were determined using the Wilcoxon signed-rank test (**p < 0.01; ***p < 0.001; ****p < 0.0001).

To investigate the effect of sorafenib treatment on the n-6 and n-3 PUFA epoxide formation, we measured the concentrations of the epoxymetabolites and corresponding dihydroxy metabolites by quantitative LC-ESI-MS/MS analysis in plasma samples of HCC patients without and during sorafenib therapy. As a result of sorafenib treatment, the levels of n-6 AA-derived epoxymetabolites 5,6-EET and 8,9-EET increased significantly. Levels of epoxymetabolites derived from the n-3 PUFAs tended to increase as well, but for DHA- and EPA-derived epoxides failed to reach significance (Figures 2A–C; Supplementary Figures S1A–C). The concentrations of EETs were higher compared to the n-3 PUFA-derived EEQs. EDP metabolite concentrations were approximately half of those observed for the EETs, while the concentrations of the EEQs were the lowest in this patient cohort.

The dihydroxy-PUFA products of the epoxy-PUFA formed *via* the sEH, respectively dihydroxyeicosatrienoic acids (DHETs) from EETs, dihydroxydocosapentaenoic acids (DiHDPAs) from EDPs and dihydroxyeicosatetraenoic acids (DiHETEs) from EEQs increased whilst on sorafenib treatment as well (Supplementary Table S1). When comparing absolute amounts of AA-, DHA- and EPA-derived epoxy-plus dihydroxy-PUFA significantly higher levels of metabolites derived from all three PUFAs were found (Figures 2D, E).

### 3.2 N-6 and n-3 fatty acid levels decrease during sorafenib treatment

To explore the presence of LC-PUFA, the fatty acid composition in plasma from patients with HCC was analyzed without and during sorafenib treatment by gas chromatography.

**FIGURE 3:**
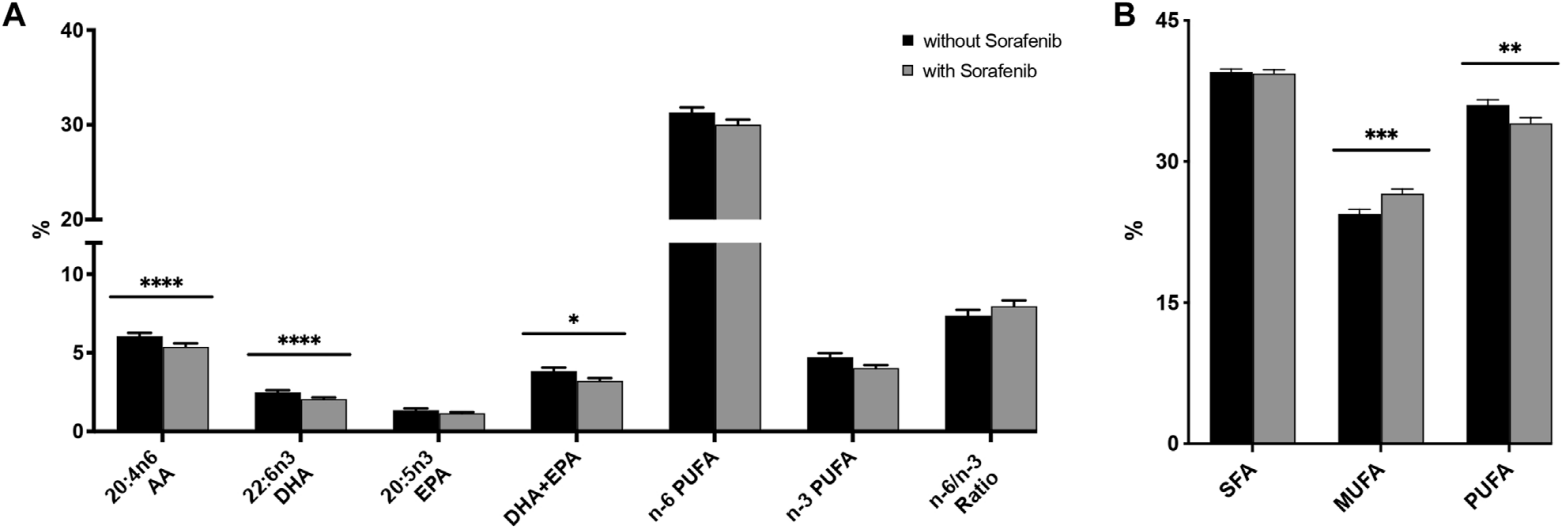
**(A)** Relative n-3 (docosahexaenoic acid, DHA; eicosapentaenoic acid, EPA) and n-6 (arachidonic acid, AA) PUFA levels in plasma from *n* = 43 patients with hepatocellular carcinoma (HCC) without and during sorafenib treatment individually, summarized and as a ratio. **(B)** Relative content of saturated fatty acids (SFA), monounsaturated fatty acids (MUFA) and polyunsaturated fatty acids (PUFA) in plasma from *n* = 43 patients with HCC without and undergoing sorafenib treatment. Statistical differences were determined using the Wilcoxon signed-rank test (*p < 0.05, **p < 0.01, ***p < 0.001, ****p < 0.0001).

Interestingly, we found a decrease in the relative content of n-6 (AA) and n-3 (DHA) PUFAs, with an increasing n-6/n-3 ratio (Figure 3A; Supplementary Table S2).

HCC patients without sorafenib had significantly higher levels of monounsaturated FAs (MUFA, *p* < 0.001)—comprising palmitoleic acid (C16:1 n-7c), oleic acid (C18:1 n-9c), and nervonic acid (C24:1 n-9)—and significantly lower levels of PUFAs (*p* < 0.01)—comprising EPA (C20:5 n-3), docosapentaenoic acid (DPA, C22:5 n-3), DHA (C22:6 n-3), linoleic acid (LA, C18: 2 n-6), dihomo-gamma-linolenic acid (DGLA, 20:3 n-6), AA (20:4 n-6), and adrenic acid (AdA, C22:4 n-6)—but there was no significant difference in the content of saturated fatty acids (SFA)—comprising myristic acid (C14:0), palmitic acid (C16:0), stearic acid (C18:0), arachidic acid (C20:0), behenic acid (C22:0), and lignoceric acid (C24:0)—(Figure 3B). With respect to specific fatty acids, HCC patients whilst on sorafenib treatment had a significantly lower relative content of the n-3 PUFA DHA (*p* < 0.0001), thus a significant decrease in DHA + EPA (*p* < 0.05), analogous to the HS-Omega-3 Index (von Schacky, 2020), and also a significant lower relative content of the n-6 PUFA AA (*p* < 0.0001).

### 3.3 N-6 and n-3 cytochrome P450 epoxy and dihydroxy product ratios do not support the hypothesis of increased sEH inhibition during sorafenib treatment

To determine whether the sEH inhibitory effect of sorafenib is detectable from the lipid metabolites assessed here, we analyzed whether sorafenib treatment increases plasma content of epoxymetabolites as compared to their dihydroxy products. As a marker for the enzyme activity in the CYP epoxygenase/sEH axis the plasma ratio of EET to DHET as characterization of the sEH inhibition has been used (Liu et al., 2009). We adapted this approach using the following equation for the AA-as well as the DHA- and EPA derived epoxy- and dihydroxy compounds:

$$sEH-Activity=\frac{dihydroxy-PUFA}{epoxy-PUFA}$$

**FIGURE 4:**
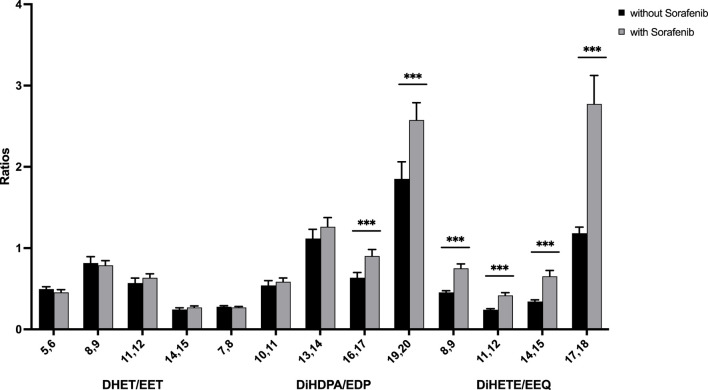
Ratio of n-6 and n-3 PUFA-derived dihydroxy to epoxy-PUFA as a marker for sEH activity in *n* = 43 patients with HCC without and undergoing sorafenib treatment (***p < 0.001).

However, in contrast to lower ratios that would indicate lower sEH activity, we found higher dihydroxy/epoxy product ratios in the sorafenib treated patients (Figure 4). This does not support lower sEH activity in the sorafenib-treated patients. Interestingly, these higher ratios were only significant for the n-3 PUFA DHA- and EPA-derived metabolites.

### 3.4 Metabolization of AA and EPA to their derived cytochrome P450 epoxy and dihydroxy products is similar, while metabolization of DHA to 19,20-EDP and 19,20-DiHDPA is markedly higher

In order to assess total CYP-epoxide and corresponding dihydroxy formation as a function of their respective substrate fatty acids we analyzed the epoxy and corresponding dihydroxy concentrations as a ratio with their respective substrate PUFA:

$$CYP-products=\frac{\left(epoxy+dihydroxy\;PUFAs\right)}{PUFAs}$$

**FIGURE 5:**
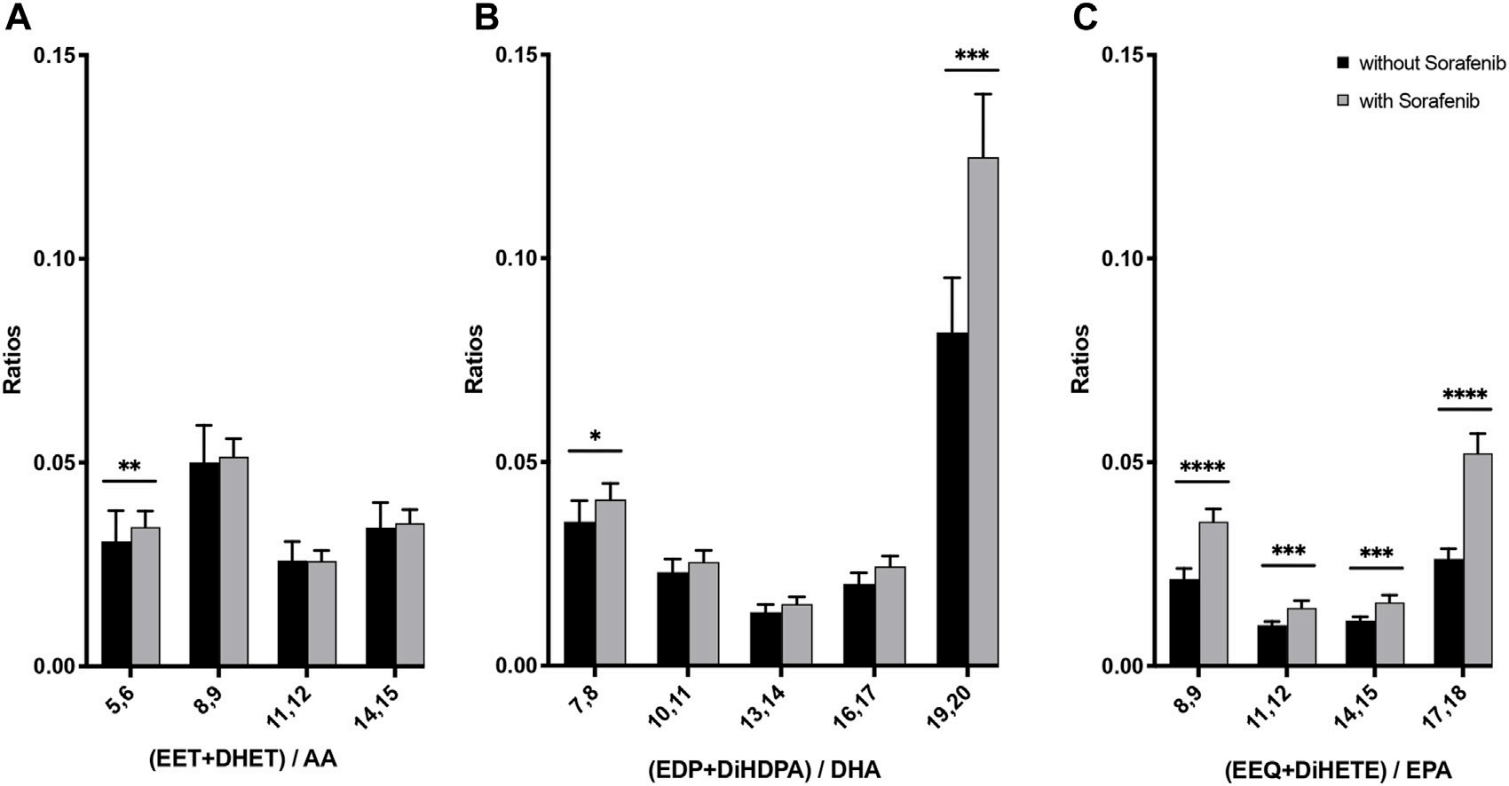
N-3 and n-6 PUFA-derived epoxides plus dihydroxy compounds as a marker for the presence of CYP metabolites in plasma from *n* = 43 patients with HCC without and undergoing sorafenib treatment. **(A)** Ratio of AA-derived products divided by AA plasma content, **(B)** ratio of DHA-derived products divided by DHA plasma content, **(C)** ratio of EPA-derived products divided by EPA plasma content (*p < 0.05, **p < 0.01; ***p < 0.001; ****p < 0.0001).

We found higher CYP product/PUFA ratios due to sorafenib treatment, providing evidence of increased presence of bioactive epoxy-PUFA from AA, EPA, and DHA in patients undergoing sorafenib treatment (Figures 5A–C). Furthermore, when analyzed as a ratio of DHA-derived CYP-products versus DHA as substrate, the 19,20-metabolites were found to be the predominant metabolites formed (Figure 5B).

## 4 Discussion

We found significant differences in plasma fatty acid composition in patients with HCC without sorafenib compared to during sorafenib treatment. Relative levels of AA and DHA were significantly lower during sorafenib treatment. Furthermore, we were able to demonstrate significantly higher EET levels and a trend towards increased n-3 CYP metabolites especially 19,20-EDP in this study population with HCC receiving sorafenib treatment.

When taking into account the different levels of the precursor n-3 PUFAs EPA and DHA we were able to establish that EPA is metabolized by CYP enzymes to a similar extent as AA, while DHA utilization was higher, leading to significantly increased levels of the 19,20-metabolites derived from DHA during sorafenib treatment (Figures 2E, 5B). Given that previous data from mouse models show inhibition of tumor angiogenesis and reduced cell invasion by increasing 19,20-EDP (Zhang et al., 2013) and to dampen and alleviate inflammation in the liver (López-Vicario et al., 2015), this could be a beneficial effect of sorafenib that could be harnessed in HCC therapy by supplementing DHA.

Generally, a beneficial role of n-3 PUFAs to dampen development of HCC is described both in animal models (Lim et al., 2009; Weylandt et al., 2011; Inoue-Yamauchi et al., 2017) and human observation studies (Sawada et al., 2012; Gao et al., 2015; Koh et al., 2016). This and the disbalance of the n-6/n-3 ratio in the Western Diet (Harris, 2006) suggests that supplementation of n-3 PUFAs could balance the n-6/n-3 ratio and may reduce tumor progression in HCC patients, regardless of sorafenib treatment. With all the described effects of n-3 PUFAs as receptor agonists, modulators of molecular signalling pathways and inflammatory responses, and data indicating that n-3 PUFA increase the efficacy of chemotherapies and consequently the overall survival of cancer patients, n-3 PUFAs can thus be considered as pharmaceutical nutrients (Bougnoux et al., 2009; Chagas et al., 2017; Paixão et al., 2017). However, in the population studied here the n-3 PUFA baseline was not associated with the overall survival (Supplementary Figure S2).

Prior studies showed that dietary increase of baseline n-3 PUFA concentrations can enhance formation of n-3 PUFA-derived CYP epoxy-PUFA (Fischer et al., 2014; Sarparast et al., 2020; Weylandt et al., 2022). Higher levels of n-3 PUFA may thus potentially increase anti-tumor n-3 PUFA-derived epoxymetabolites as well as decrease pro-tumor n-6 PUFA-derived metabolites (Zhang et al., 2014). Interestingly, in this study we found lower levels of DHA in patients treated with sorafenib (Figure 3), further supporting the concept to increase DHA in the daily diet in order to increase also levels of 19,20-EDP in HCC patients treated with sorafenib.

Many classes of currently used drugs can block or modify pathways of lipid mediator formation. Particularly well-established are non-steroidal anti-inflammatory drugs inhibiting the cyclooxygenase (COX) enzymes as well as numerous clinically well-established substances that modify (induce, inhibit) CYP enzymes and thereby modify lipid mediator formation. In general, by using quantitative LC-MS/MS oxylipin analysis in the context of established pharmacotherapy (pharmacolipidomics) as shown in this paper we hope to identify oxylipins that might be used to stratify and possibly also modify and improve response to treatments: Experimental data indicate strong biological effects of specific lipid mediators, particularly with regard to inflammation-dampening oxylipins from n-3 PUFA (n-3 IDOs) (Weylandt et al., 2022) in contrast to often inflammation-promoting oxylipins from n-6 PUFA (n-6 IPOs). As concentrations of these can be modified by changes in fatty acids substrates, as well as established drugs such as sorafenib, there could be a rationale for targeted modifications of the n-6/n-3 PUFA ratio in the diet in the context of established pharmacotherapy to harness these effects.

In systemic HCC therapy, the combination of immune checkpoint inhibitors, and VEGF pathway inhibitors such as sorafenib could promote an immune-permissive environment, thereby enhancing the response to immune therapeutic approaches. Immunotherapy is effective in only approximately 1/3 of cancer patients, and targeting the TME to decrease tumor cell evasion is regarded as an opportunity to improve response to immunotherapy, as conversion of “cold” tumors to “hot” tumors with T cell infiltration is associated with a better response rate to cancer treatment (Bonaventura et al., 2019). TME-modifying properties of lipid mediator levels may therefore enhance antitumor effects by transforming the immune landscape. Aspirin dampens tissue inflammation *via* the metabolism of arachidonic acid in the COX enzymatic pathways and can reduce cellular growth in hepatocellular carcinoma (Tao et al., 2018; Refolo et al., 2020). Whether a CYP/sEH-dependent effect on lipid mediators—which could be modulated/enhanced by sorafenib as described here—could also play a role in the TME, remains a topic for future studies that are directly analyzing effects of n-3 IDO and n-6 IPO levels and formation, as well as immune cells in liver and liver tumor tissue.

In our data presented here, we were not able to discern an sEH inhibitory effect of sorafenib (Figure 4). Non-etheless we established significantly higher levels of CYP-derived epoxy and dihydroxy metabolites in patients undergoing sorafenib treatment. While previous results from one of us show that storage at −80°C should be sufficient to yield stable CYP derived oxylipin readings comparable to other oxylipins (Gladine et al., 2019; Koch et al., 2020), there might have been variations in the process of blood sampling and storage leading to changes in epoxy-PUFA levels. Another explanation would be that sorafenib may have more complex effects on PUFA-derived metabolites in humans, with increased formation of epoxy-PUFAs. We did not analyse expression of sEH directly, therefore there is a possibility of increased sEH expression, as described in an animal models of high fat diet induced liver disease (López-Vicario et al., 2015) which might compensate for an inhibitory effect of sorafenib. Interestingly, in our pilot study we found an increase of 8,9-EET, 11,12-EET and 14,15-EET levels in HCC patients treated with sorafenib (Leineweber et al., 2020) while we found a significant increase only of 5,6-EET and 8,9-EET levels here. We believe this might be due to the analytical limitations due to differences in sample taking and storage, and not a mechanistic difference in the effect observed. Indeed, we suggest to use the combined analysis of epoxides and dihydroxy compounds (as in Figures 2D–F) to assess epoxy metabolite formation. However future studies could better address this question by analyzing the samples under defined conditions and at different time intervals after blood drawing to measure the effects of these variations on epoxy- and dihydroxy-PUFA levels.

## 5 Conclusion

In this study, we investigated the effect of sorafenib treatment on PUFA formation and epoxy lipid mediator concentrations in peripheral blood plasma in a group of 43 HCC patients as a sub-analysis of the randomized, controlled, multicenter phase II SORAMIC study. We were able to demonstrate markedly increased epoxy plus dihydroxy PUFA concentrations in the peripheral blood of HCC patients undergoing sorafenib therapy. These results support previous findings that sorafenib treatment induces a change in epoxy-/dihydroxy-PUFA concentrations.

Given the anti-tumor effects described in experimental models for the n-3 PUFA-derived 19,20-EDP, these data further support the hypothesis that dietary n-3 PUFA supplementation in addition to sorafenib treatment could contribute anti-tumor effects due to n-3 epoxy-PUFA.

## Data Availability

The raw data supporting the conclusion of this article will be made available by the authors, without undue reservation.

## Glossary

AA: arachidonic acid
AFP: alpha-fetoprotein
AlBi: albumin-bilirubin
BCLC: barcelona clinic liver cancer
BHT: butylated hydroxytoluene
BMI: body mass index
COX: cyclooxygenase
CT: computer tomography
CYP: cytochrome P450
DHA: docosahexaenoic acid
DHET: dihydroxyeicosatrienoic acid
DiHDPA: dihydroxydocosapentaenoic acid
DiHETE: dihydroxyeicosatetraenoic acid
EDP: epoxydocosapentaenoic acid
EEQ: epoxyeicosatetraenoic acid
EET: epoxyeicosatrienoic acid
EGF: epidermal growth factor
EPA: eicosapentaenoic acid
FGF: fibroblast growth factor
FID: flame ionization detector
GC: gas chromatography
HCC: hepatocellular carcinoma
HPLC/ESI: high performance liquid chromatography electrospray ionization
LC-PUFA: long-chain polyunsaturated fatty acid
mEH: microsomal epoxide hydrolase
MRI: magnetic resonance imaging
MS: mass spectrometry
NAFLD: fatty acid non-alcoholic fatty liver disease
NASH: non-alcoholic steatohepatitis
NF-κB: nuclear factor kappa B
n-3 PUFA: omega-3 polyunsaturated fatty acids
n-6 PUFA: omega-6 polyunsaturated fatty acids
OS: overall survival
PDA: pentadecanoic acid
PDGFR-β: platelet-derived growth factor receptor β
PFS: progression-free survival
RAF: rapidly accelerated fibrosarcoma
sEH: soluble epoxide hydrolase
SIRT: selective internal radiation treatment
SPE: solid phase extraction
VEGFR: vascular endothelial growth factor receptor
