# Corrigendum: Sorafenib increases cytochrome P450 lipid metabolites in patient with hepatocellular carcinoma

**DOI:** 10.3389/fphar.2023.1354581

**Published:** 2024-01-11

**Authors:** Can G. Leineweber, Miriam Rabehl, Anne Pietzner, Nadine Rohwer, Michael Rothe, Maciej Pech, Bruno Sangro, Rohini Sharma, Chris Verslype, Bristi Basu, Christian Sengel, Jens Ricke, Nils Helge Schebb, Karsten-H. Weylandt, Julia Benckert

**Affiliations:** ^1^ Medical Department B, Division of Hepatology, Gastroenterology, Oncology, Hematology, Palliative Care, Endocrinology, and Diabetes, Brandenburg Medical School, University Hospital Ruppin-Brandenburg, Neuruppin, Germany; ^2^ Faculty of Health Sciences, Joint Faculty of the Brandenburg University of Technology, Brandenburg Medical School and University of Potsdam, Potsdam, Germany; ^3^ Institut d’Investigacions Biomèdiques August Pi i Sunyer (IDIBAPS), Barcelona, Spain; ^4^ Department of Molecular Toxicology, German Institute of Human Nutrition Potsdam-Rehbruecke, Nuthetal, Germany; ^5^ Lipidomix, Berlin, Germany; ^6^ Department of Radiology and Nuclear Medicine, Otto-von-Guericke University, Magdeburg, Germany; ^7^ Liver Unit and HPB Oncology Area, Clinica Universidad de Navarra and CIBEREHD, Pamplona, Spain; ^8^ Department of Surgery and Cancer, Imperial College London, London, United Kingdom; ^9^ Department of Digestive Oncology, University Hospitals Leuven, Leuven, Belgium; ^10^ Department of Oncology, University of Cambridge, Cambridge, United Kingdom; ^11^ Radiology Department, Grenoble University Hospital, La Tronche, France; ^12^ Department of Radiology, University Hospital, Ludwig-Maximilians-University (LMU) Munich, Munich, Germany; ^13^ Chair of Food Chemistry, Faculty of Mathematics and Natural Science, University of Wuppertal, Wuppertal, Germany; ^14^ Department of Hepatology and Gastroenterology, Charité—Universitätsmedizin Berlin, Corporate Member of Freie Universität Berlin and Humboldt—Universität zu Berlin, Berlin, Germany

**Keywords:** hepatocellular carcinoma, cytochrome P450, sorafenib, EET, EDP, omega-3 fatty acids, oxylipins, lipidomics

In the published article, there was an error in Figures 2, 3, 5 as published. In an earlier version of the article, the asterisks in the figures mentioned above were not displayed as a visualization of the *p*-values. The corrected Figures and their unchanged captions appear below.

**FIGURE 2:**
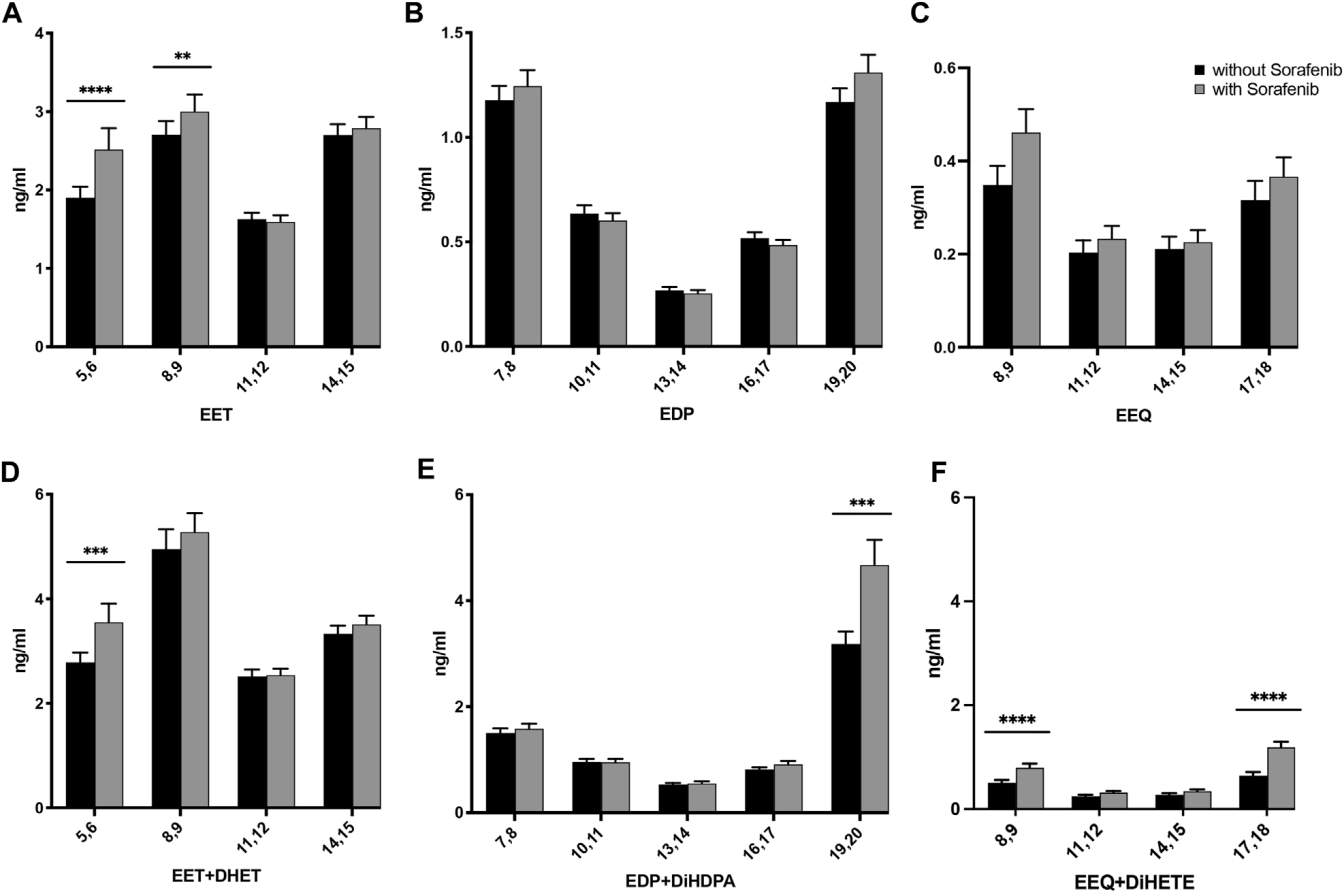
Effects on the concentrations of **(A)** AA-, **(B)** DHA-, and **(C)** EPA-derived epoxy-PUFA EETs, EDPs, and EEQs; and **(D)** AA-derived epoxy-PUFA plus dihydroxy-PUFA, **(E)** DHA-derived epoxy-PUFA plus dihydroxy-PUFA, and **(F)** EPA-derived epoxy-PUFA plus dihydroxy-PUFA in the plasma of *n* = 43 patients with hepatocellular carcinoma (HCC) without and undergoing sorafenib treatment (ng/mL ± standard error of the mean). Statistical differences were determined using the Wilcoxon signed-rank test (**p < 0.01; ***p < 0.001; ****p < 0.0001).

**FIGURE 3:**
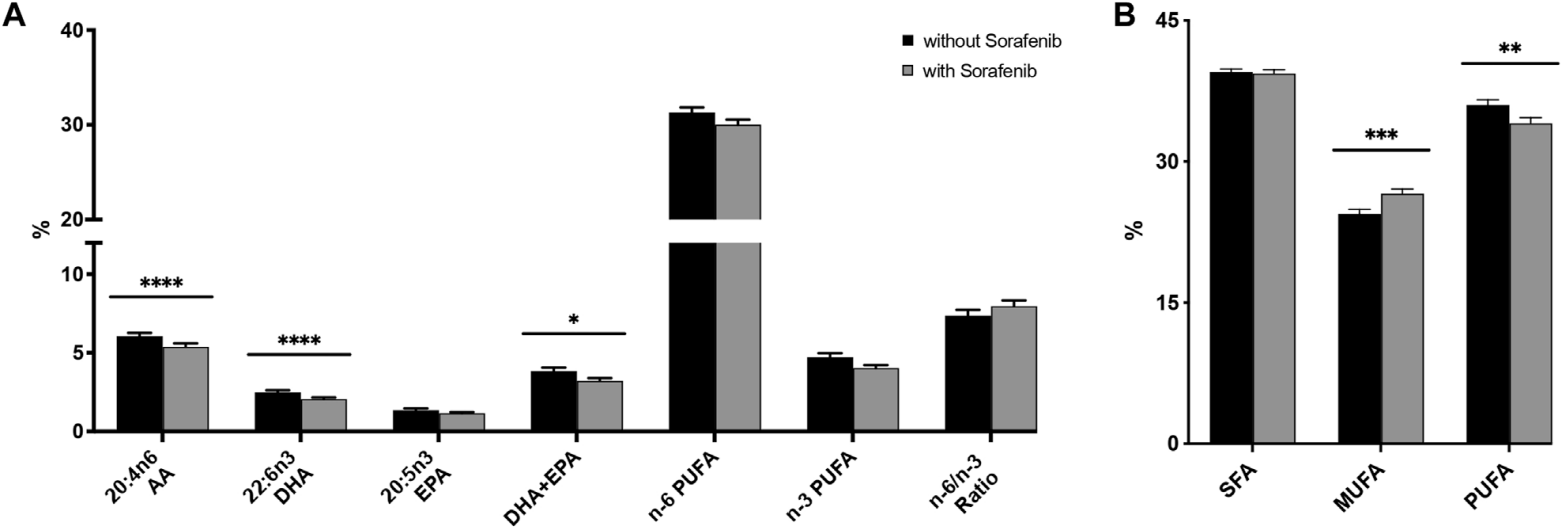
**(A)** Relative n-3 (docosahexaenoic acid, DHA; eicosapentaenoic acid, EPA) and n-6 (arachidonic acid, AA) PUFA levels in plasma from *n* = 43 patients with hepatocellular carcinoma (HCC) without and during sorafenib treatment individually, summarized and as a ratio. **(B)** Relative content of saturated fatty acids (SFA), monounsaturated fatty acids (MUFA) and polyunsaturated fatty acids (PUFA) in plasma from *n* = 43 patients with HCC without and undergoing sorafenib treatment. Statistical differences were determined using the Wilcoxon signed-rank test (*p < 0.05, **p < 0.01, ***p < 0.001, ****p < 0.0001).

**FIGURE 5:**
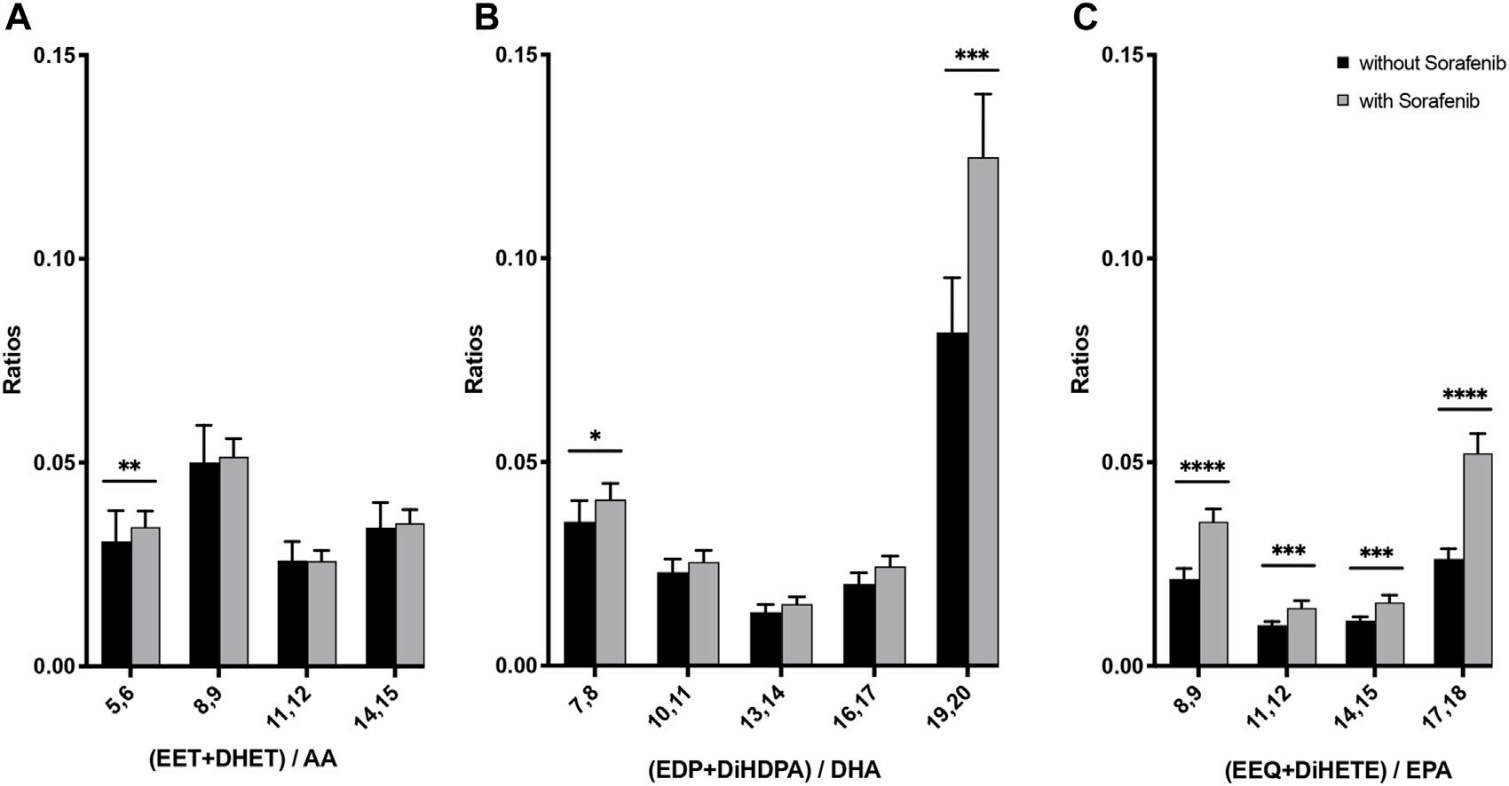
N-3 and n-6 PUFA-derived epoxides plus dihydroxy compounds as a marker for the presence of CYP metabolites in plasma from *n* = 43 patients with HCC without and undergoing sorafenib treatment. **(A)** Ratio of AA-derived products divided by AA plasma content, **(B)** ratio of DHA-derived products divided by DHA plasma content, **(C)** ratio of EPA-derived products divided by EPA plasma content (*p < 0.05, **p < 0.01; ***p < 0.001; ****p < 0.0001).

In the published article, there was an error in the **Funding** statement. The correct Funding statement appears below:

The author(s) declare financial support was received for publication of this article. Publication was funded by the Brandenburg Medical School (Medizinische Hochschule Brandenburg, MHB) publication fund supported by the German Research Foundation (Deutsche Forschungsgemeinschaft, DFG) and by the Ministry of Science, Research and Culture of the State of Brandenburg.

In the published article, there was a typographical error. A correction has been made to the **Introduction**. The original sentence stated:

“These epoxymetabolites are then further metabolized via she into their biologically less active corresponding dihydroxy metabolites”

The corrected sentence appears below:

“These epoxymetabolites are then further metabolized via sEH into their biologically less active corresponding dihydroxy metabolites”

The authors apologize for these errors and state that this does not change the scientific conclusions of the article in any way. The original article has been updated.

