# Supplementary material for: Sorafenib increases cytochrome P450 lipid metabolites in patient with hepatocellular carcinoma
Source: Front Pharmacol. 2023 Mar 3;14:1124214. doi: 10.3389/fphar.2023.1124214 (PMC10020374; doi:10.3389/fphar.2023.1124214)
Supplement: Supplementary file 1 [file DataSheet1.PDF]

Supplementary Material

Sorafenib increases Cytochrome P450 Lipid Metabolites in Patients with Hepatocellular Carcinoma

Can G. Leineweber [1,2,3], Miriam Rabehl[1,2], Anne Pietzner[1,2], Nadine Rohwer[1,2,4], Michael Rothe[5], Maciej Pech[6], Bruno Sangro[7], Rohini Sharma[8], Chris Verslype[9], Bristi Basu[10], Christian Sengel[11], Jens Ricke[12], Nils Helge Schebb[13], Karsten-H. Weylandt[1,2*#], Julia Benckert[14#]

*** Correspondence:** karsten.weylandt@mhb-fontane.de

1 Supplementary Figures and Tables

1.1 Supplementary Figures

Supplementary Figure 1

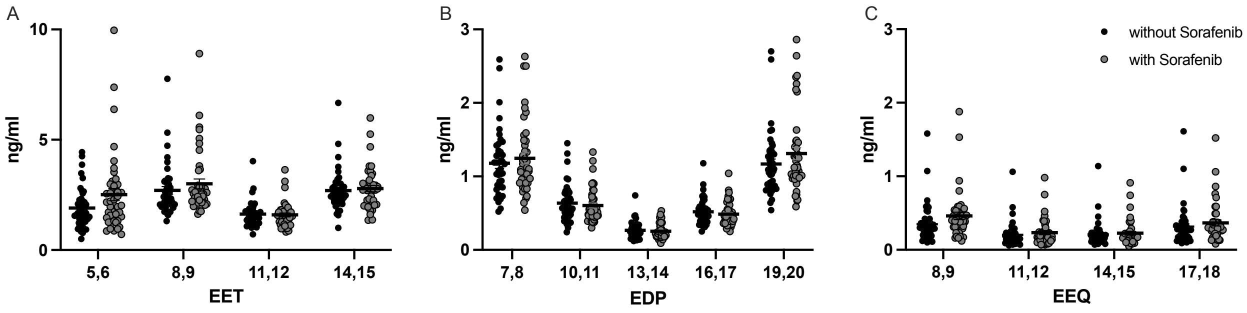

Supplementary Figure 1 Effects on the concentrations of (A) AA-, (B) DHA-, and (C) EPA-derived epoxy-PUFA EETs, EDPs, and EEQs in the plasma of *n* = 43 patients with hepatocellular carcinoma (HCC) without and with sorafenib treatment (ng/mL + standard error of the mean) as interleaved scatter.

Supplementary Figure 2

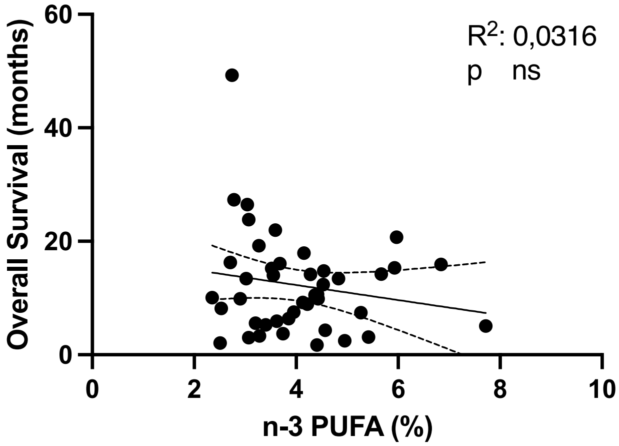

Supplementary Figure 2 Correlation of the relative n-3 PUFA baseline to the overall survival in n = 43 patients with HCC analyzed in this study. In this study population the n-3 PUFA baseline was not associated with the overall survival.

1.2 Supplementary Tables

Supplementary Table 1. Epoxy and corresponding dihydroxy metabolites in blood samples.

	**Without sorafenib**		**With sorafenib**		
	**ng/ml**	**SEM**	**ng/ml**	**SEM**	
14,15-EET	**2,70**	0,14	**2,79**	0,14	
11,12-EET	**1,63**	0,08	**1,59**	0,09	
8,9-EET	**2,70**	0,18	**3,00**	0,22	**
5,6-EET	**1,90**	0,14	**2,52**	0,27	****
14,15-DHET	**0,63**	0,06	**0,72**	0,06	
11,12-DHET	**0,89**	0,09	**0,95**	0,08	
8,9-DHET	**2,25**	0,26	**2,28**	0,19	
5,6-DHET	**0,88**	0,06	**1,03**	0,10	
19,20-EDP	**1,17**	0,07	**1,31**	0,08	
16,17-EDP	**0,52**	0,03	**0,49**	0,02	
13,14-EDP	**0,27**	0,02	**0,25**	0,02	
10,11-EDP	**0,64**	0,04	**0,60**	0,03	
7,8-EDP	**1,18**	0,07	**1,25**	0,08	
19,20-DiHDPA	**2,01**	0,22	**3,36**	0,42	***
16,17-DiHDPA	**0,30**	0,03	**0,43**	0,05	***
13,14-DiHDPA	**0,26**	0,02	**0,30**	0,03	
10,11-DiHDPA	**0,32**	0,04	**0,35**	0,04	

7,8-DiHDPA	**0,33**	0,02	**0,34**	0,03	
17,18-EEQ	**0,32**	0,04	**0,37**	0,04	
14,15-EEQ	**0,21**	0,03	**0,23**	0,03	
11,12-EEQ	**0,20**	0,03	**0,23**	0,03	
8,9-EEQ	**0,35**	0,04	**0,46**	0,05	**
17,18-DiHETE	**0,33**	0,03	**0,82**	0,09	****
14,15-DiHETE	**0,06**	0,01	**0,12**	0,01	****
11,12-DiHETE	**0,04**	0,00	**0,08**	0,01	****
8,9-DiHETE	**0,16**	0,02	**0,34**	0,03	****

Shown are the means in ng/ml ± SEM of lipid mediator formation in n=43 patients with hepatocellular carcinoma without and with sorafenib treatment. Statistical differences were determined using Wilcoxon signed-rank test (** p<0,01; *** p<0,001; **** p<0,0001).

Supplementary Table 2. Absolute and relative n-6 and n-3 PUFA values.

	**Absolute PUFA values**				**Relative PUFA values**				
	**Without sorafenib**		**With sorafenib**		**Without sorafenib**		**With sorafenib**		
	**µg/ml**	**SEM**	**µg/ml**	**SEM**	**%**	**SEM**	**%**	**SEM**	
C14:0	**37,41**	3,25	**43,15**	4,21	**1,76**	0,10	**1,95**	0,14	
C16:0	**558,60**	31,76	**598,50**	46,57	**26,06**	0,30	**26,40**	0,35	
C16:1n7	**50,07**	3,97	**56,59**	4,49	**2,40**	0,16	**2,60**	0,17	
C18:0	**188,12**	10,04	**187,98**	14,38	**8,81**	0,15	**8,34**	0,16	
C18:1n9c	**445,69**	26,31	**507,44**	35,69	**20,77**	0,39	**22,70**	0,42	***

C18:2n6c	**489,08**	29,92	**501,93**	38,59	**22,67**	0,56	**22,33**	0,49	
C20:0	**16,18**	0,53	**16,07**	0,56	**0,86**	0,07	**0,80**	0,04	
C22:0	**18,25**	0,52	**17,02**	0,50	**0,96**	0,07	**0,85**	0,04	
C20:3n6	**34,95**	1,97	**32,47**	2,04	**1,69**	0,07	**1,49**	0,05	
C20:4n6	**127,80**	6,88	**118,61**	8,11	**6,06**	0,22	**5,39**	0,23	****
C20:5n3	**27,31**	3,01	**24,41**	1,78	**1,35**	0,12	**1,16**	0,08	
C24:0	**19,48**	0,61	**19,45**	0,54	**1,07**	0,10	**1,01**	0,08	
C24:1n9	**26,11**	1,56	**28,40**	2,40	**1,29**	0,07	**1,27**	0,05	
C22:4n6	**16,78**	0,68	**16,64**	0,69	**0,88**	0,07	**0,81**	0,04	
C22:5n3	**16,85**	0,81	**16,70**	0,73	**0,88**	0,07	**0,81**	0,04	
C22:6n3	**52,72**	4,06	**46,91**	3,91	**2,50**	0,13	**2,07**	0,10	****
SFA	**838,04**	44,32	**882,17**	63,34	**39,53**	0,32	**39,36**	0,40	
MUFA	**521,87**	30,06	**592,43**	40,48	**24,45**	0,46	**26,58**	0,50	****
PUFA	**765,49**	40,45	**757,66**	51,48	**36,02**	0,55	**34,06**	0,61	**
n-6 PUFA	**668,61**	36,62	**669,64**	47,25	**31,30**	0,55	**30,02**	0,56	
n-3 PUFA	**96,88**	7,35	**88,02**	5,90	**4,72**	0,27	**4,05**	0,18	
EPA+DHA	**80,03**	6,66	**71,32**	5,32	**3,84**	0,23	**3,23**	0,16	
n-6/3 PUFA	**7,37**	0,38	**7,98**	0,36	**7,37**	0,38	**7,98**	0,36	

Shown are the means in ng/ml ± SEM of lipid mediator formation in n=43 patients with hepatocellular carcinoma without and with sorafenib treatment. Statistical differences were determined using Wilcoxon signed-rank test (** p<0,01; *** p<0,001; **** p<0,0001).
